# Combined Aerobic Exercise with Intermittent Fasting Is Effective for Reducing mTOR and Bcl-2 Levels in Obese Females

**DOI:** 10.3390/sports12050116

**Published:** 2024-04-25

**Authors:** Purwo Sri Rejeki, Adi Pranoto, Deandra Maharani Widiatmaja, Dita Mega Utami, Nabilah Izzatunnisa, Sugiharto, Ronny Lesmana, Shariff Halim

**Affiliations:** 1 Physiology Division, Department of Medical Physiology and Biochemistry, Faculty of Medicine, Universitas Airlangga, Surabaya 60132, East Java, Indonesia; 2 Doctoral Program of Medical Science, Faculty of Medicine, Universitas Airlangga, Surabaya 60132, East Java, Indonesia; adi.pranoto-2020@fk.unair.ac.id; 3 Medical Program, Faculty of Medicine, Universitas Airlangga, Surabaya 60132, East Java, Indonesia; dean.maharani.widiatmaja-2021@fk.unair.ac.id (D.M.W.); dita.mega.utami-2020@fk.unair.ac.id (D.M.U.); nabilah.izzatunnisa-2019@fk.unair.ac.id (N.I.); 4 Department of Sport Science, Faculty of Sport Science, Universitas Negeri Malang, Malang 65145, East Java, Indonesia; sugiharto@um.ac.id; 5 Physiology Division, Department of Biomedical Science, Faculty of Medicine, Universitas Padjajaran, Bandung 45363, West Java, Indonesia; ronny@unpad.ac.id; 6 Faculty of Health Sciences, University Technology MARA (UiTM) Pulau Pinang, Bertam Campus, Kepala Batas 13200, Pulau Pinang, Malaysia; halimshariff@uitm.edu.my

**Keywords:** aerobic exercise, Bcl-2, intermittent fasting, mTOR, obesity

## Abstract

The integration of combined aerobic exercise and intermittent fasting (IF) has emerged as a strategy for the prevention and management of obesity, including its associated health issues such as age-related metabolic diseases. This study aimed to examine the potential of combined aerobic exercise and IF as a preventative strategy against cellular senescence by targeting mTOR and Bcl-2 levels in obese females. A total of 30 obese women, aged 23.56 ± 1.83 years, body fat percentage (FAT) 45.21 ± 3.73% (very high category), BMI 30.09 ± 3.74 kg/m^2^ were recruited and participated in three different types of interventions: intermittent fasting (IF), exercise (EXG), and a combination of intermittent fasting and exercise (IFEXG). The intervention program was carried out 5x/week for 2 weeks. We examined mTOR and Bcl-2 levels using ELISA kits. Statistical analysis used the one-way ANOVA test and continued with Tukey’s HSD post hoc test, with a significance level of 5%. The study results showed that a combination of aerobic exercise and IF significantly decreased mTOR levels (−1.26 ± 0.79 ng/mL) compared to the control group (−0.08 ± 1.33 ng/mL; *p* ≤ 0.05). However, combined aerobic exercise and IF did not affect Bcl-2 levels significantly (−0.07 ± 0.09 ng/mL) compared to the control group (0.01 ± 0.17 ng/mL, *p* ≥ 0.05). The IF-only group, exercise-only group, and combined group all showed a significant decrease in body weight and fat mass compared to the control group (*p* ≤ 0.05). However, the combined aerobic exercise and IF program had a significant effect in reducing the total percentage of body fat and fat mass compared to the IF-only group (*p* ≤ 0.05). Therefore, it was concluded that the combined intermittent fasting and exercise group (IFEXG) undertook the most effective intervention of the three in terms of preventing cellular senescence, as demonstrated by decreases in the mTOR level, body weight, and fat mass. However, the IFEXG did not present reduced Bcl-2 levels.

## 1. Introduction

Obesity is characterized by the abnormal or excessive accumulation of fat, posing potential health risks. The Body Mass Index (BMI), a widely used indicator of weight relative to height, is a fundamental tool for classifying overweight and obesity in adults. However, the BMI has several limitations because it does not account for factors such as muscle mass, bone density, or body composition. Nonetheless, despite those limitations, the BMI is widely used due to its simplicity and ease of use in population-based studies and public health interventions. According to World Health Organization (WHO) criteria for adults, a BMI greater than or equal to 25 indicates overweight, while a BMI greater than or equal to 30 signifies obesity. In 2016, the WHO estimated that over 650 million adults, constituting 13% of the global adult population, were affected by obesity, underscoring the pressing significance of this public health concern [1]. In Indonesia, according to the 2018 Basic Health Survey (Riskesdas) data, the prevalence of obesity stands at 21.8%, indicating a twofold increase from 2007 (10.5%) [2]. A study by Ayuningtyas et al. [3] further reveals that the highest prevalence of obesity in Indonesia is observed among females (26.4%) and adults aged 25–59 years (24.8%). The development of obesity involves both an increase in fat cell size (adipocyte hypertrophy) and number (adipocyte hyperplasia). Adipocyte enlargement is the primary cause of metabolic dysfunction associated with obesity, leading to chronic low-grade inflammation. This inflammation, along with increased circulating fatty acids, contributes to conditions like insulin resistance, cardiovascular disease, a higher risk of neurodegenerative disorders, and certain cancers in obese individuals. Altered metabolic regulation, insulin resistance, inflammation, and compromised immune function are also associated with aging. The simultaneous occurrence of similar immunological profiles in obesity and aging suggests a potential link between the two processes, implying that obesity may accelerate aging and the onset of age-related diseases [4]. This can affect both health and the economy by reducing work productivity and increasing healthcare costs linked to obesity-related health issues and early aging [5].

Obesity results from an imbalance in energy homeostasis, where energy intake exceeds energy expenditure [6]. Physical exercise has been used as a crucial tool in preventing and treating overweight and obesity [7,8,9]. Moderate-intensity exercise has been shown to reduce the levels of proinflammatory cytokines such as IL-6 and TNF-α in young adult females with obesity [10]. Current guidelines suggest at least 150 min of moderate aerobic activity or 75 min of vigorous aerobic activity per week, emphasizing the linear relationship between energy expenditure and exercise intensity. A marker of an individual entering vigorous aerobic exercise is the inability to utter more than a few words without pausing momentarily to inhale. Some examples of activities that are classified as vigorous intensity include swimming laps, running, hiking, playing football, basketball, or soccer, and jumping rope. Regular exercise plays a significant role in regulating blood sugar and enhancing skeletal muscles’ ability to absorb glucose, ultimately contributing to the prevention of metabolic diseases and improving the metabolic phenotype of various tissues [11]. Intermittent fasting (IF) refers to scheduled periods of minimal or no caloric intake, typically including a daily 16 h fast, a 24 h fast on alternate days, or fasting for two non-consecutive days per week. Caloric consumption during these fasting periods usually varies between 0 and 25% of caloric needs [12]. Some previous studies showed that IF had better participant compliance and was more beneficial in reducing fat mass (FM) [13,14]. The beneficial effects of intermittent fasting (IF) primarily result from weight reduction. IF induces a metabolic switch, optimizing cellular fuel utilization, and favoring ketone bodies and fatty acids, thereby enhancing metabolic flexibility and improving mitochondrial function. Additionally, IF activates autophagy, defends against oxidative and metabolic stress, and suppresses inflammation [15]. In humans, the cycle of fasting and refeeding brings about beneficial effects on factors associated with aging, diabetes, autoimmunity, cardiovascular disease, neurodegeneration, and cancer risk [16].

B-cell lymphoma 2 (Bcl-2) is a key anti-apoptotic protein in the Bcl-2 family [17]. Within healthy cells, the anti-apoptotic Bcl-2 proteins prevent the action of apoptosis effectors, namely BAX and BAK, in order to protect the cells and ensure their survival [18]. Bcl-2 exerts its anti-apoptotic function by forming a heterodimer with BAX, regulating the Ca2+ concentration, providing antioxidant effects, and inhibiting caspase activities. Its increased expression and phosphorylation are involved in controlling cell proliferation, holding significant importance in both tumor development and resistance to multiple drugs [17]. The study by Koohshoori et al. [19] showed that 4 weeks of aerobic exercise reduced Bcl-2 gene expression and protein, which might be caused by IL-6 reduction. Similar to aerobic exercise, the previous study also showed that IF reduced Bcl-2 expression in rats without acute kidney injury (AKI), although not significantly [20]. The mechanistic target of rapamycin (mTOR) is a widely preserved serine/threonine protein kinase that combines signals from environmental nutrients, growth factors, cellular energy levels, and other cellular cues to influence diverse processes including anabolism and autophagy [21]. The expression of phosphorylated mTOR (p-mTOR) was identified in a distinct epithelial region within internal organs, including the intestine, stomach, liver, pancreas, kidneys, and bladder [22]. Recent studies have discovered that the mTOR signaling pathway is crucial in regulating various aspects of adipose tissue function, including adipogenesis, lipid metabolism, thermogenesis, and adipokine synthesis/secretion. Dysregulation in mTOR signaling is associated with several conditions including obesity, type 2 diabetes, cancer, and aging [23]. In the feeding state of IF, mTOR is activated. Meanwhile, fasting triggers stress-responsive pathways like forkhead box O (FOXO) and AMP-activated protein kinase (AMPK), suppressing mTOR signaling, thereby promoting autophagy and oxidative defense mechanisms [15]. Aerobic exercise also impacts mTOR via AMPK activation, initiated by changes in the ATP/ADP ratio. The decline in ATP levels during exercise elevates AMP, activating AMPK, which, in turn, lowers mTOR levels, leading to the induction of cellular autophagy [24].

Based on these data, it is known that calorie restriction and exercise have the potential to regulate cellular senescence and have the opportunity to be studied further. However, the mechanism of combined aerobic exercise with intermittent fasting in reducing mTOR and Bcl-2 levels in obese females has never been explored. This study aimed to explore the potential of combined IF and exercise as a preventative strategy against cellular senescence by examining Bcl-2 and mTOR. Thus, this study can serve as a basic reference for developing therapies aimed at promoting quality healthy aging.

## 2. Materials and Methods

### 2.1. Experimental Design

This research was true experimental with a pretest–posttest control group design. Inclusion criteria in this study included obese women (based on Asia-Pacific BMI classification), aged 23.56 ± 1.83 years, body fat percentage (FAT) 45.21 ± 3.73% (very high category), BMI 30.09 ± 3.74 kg/m^2^, fasting blood glucose 95.18 ± 2.50 mg/dL, hemoglobin (Hb) 13.98 ± 1.58 g/dL, blood pressure (systolic 116.06 ± 1.67 mmHg, diastolic 72.86 ± 1.19 mmHg), resting heart rate (RHR) 78.30 ± 1.23 bpm, and a low level of physical activity based on the Global Physical Activity Questionnaire (GPAQ) with a score of ≤600 METs/minute/week. Subjects were randomly divided into four groups, namely CG (control group), IFG (intermittent fasting group), EXG (exercise group), and IFEXG (intermittent fasting and exercise group), each with n = 10. The calculation of the number of participants was made using the Higgins and Kleinbaum formula [25], with reference values from previous similar studies so that a minimum sample size was obtained, and 40 participants was the result [26]. The intervention was carried out five times per week for two weeks. After the two-week intervention program, anthropometric measurements, body fat, and blood samples were collected. In this study, there was a dropout rate of 0%, and the arrival rate was 100%.

### 2.2. Intermittent Fasting Protocol

Intermittent fasting (IF) consists of 16:8 (8 h eating and 16 h fasting); while undergoing IF, subjects are allowed to consume water according to each individual’s daily needs [27]. The fasting protocol was repeated five times weekly (Monday, Tuesday, Wednesday, Thursday, and Saturday) for two weeks. Subjects recorded food during the intervention period using the estimated food record method. Subjects were given a Food Photo Book published by the Ministry of Health of the Republic of Indonesia to make it easier for subjects to estimate the weight of food consumed visually. Before the research began, subjects received an explanation and training on recording their food intake and using the Food Photo Book.

### 2.3. Aerobic Exercise Protocol

Aerobic exercise was carried out using a treadmill with moderate-intensity 60–70% HRmax [7]. In the combination group with IF, exercise was carried out 30 min before breaking the fast for 40 min (5 min warm-up (50–60% HRmax), 30 min core training (continuous with 60–70% HRmax, and 5 min cool-down (50–60% HRmax)), with a frequency of 5x/week, namely Monday, Tuesday, Wednesday, Thursday, and Saturday, for two weeks [27]. Before the exercise began, their health profile was checked in terms of blood pressure, resting heart rate, oxygen saturation, and body temperature to determine whether the subject was fit for the exercise program. Training intensity was controlled using the Polar H10 heart sensor. The environment of the research room was a humidity level of 50–70% and a temperature of 26 ± 1 °C.

### 2.4. Body Composition Assessment

Body composition was measured during the pretest and posttest using the TANITA Body Composition Analyzer DC3607601(2)-1604 FA (TANITA Corporation of America, Inc., Arlington Heights, IL, USA) [28,29,30]. Data collected were weight, body fat percentage (FAT), fat mass (FM), fat-free body mass (FFM), muscle mass (MM), and total body water (TBW), as well as body mass index (BMI).

### 2.5. Blood Sampling and Biochemical Analysis

A blood sample of 4 mL was taken from the cubital vein using the BD VACUTAINER Flashback Blood Collection Needle (22 G). The blood taken was placed in a vacutainer and then centrifuged for 15 min at a speed of 3000 rpm. The serum was separated and immediately analyzed for mTOR and Bcl-2 levels. Measurement of mTOR levels used Human mTOR (Mammalian Target of Rapamycin) ELISA kits (Cat.No.: E-EL-H1655; Elabscience Biotechnology Inc., Houston, TX, USA) with an mTOR sensitivity level of 0.10 ng/mL and detection range of 0.16–10 ng/mL. Measurement of Bcl-2 levels used Human Bcl-2 (B-cell leukemia/lymphoma 2) ELISA kits (Cat.No.:E-EL-H0114; Elabscience Biotechnology Inc., Houston, TX, USA) with a Bcl-2 sensitivity level of 0.10 ng/mL and a detection range of 0.16–10 ng/mL. Several previous studies have validated the accuracy of the ELISA kits [31,32].

### 2.6. Statistical Analysis

The normality was assessed using the Shapiro–Wilk test, and homogeneity was investigated using the Levene test. The difference was examined with a paired-sample *t*-test and one-way ANOVA and continued with Tukey’s Honest Significant Difference (HSD) post hoc test. Meanwhile, data that were not normally distributed were tested using the Kruskal–Wallis and Mann–Whitney U tests. The Pearson product-moment correlation test determined the relationship of mTOR and Bcl-2 with body fat and metabolic age. All data were presented as mean ± standard deviation (SD). All statistical analyses used a significance level (*p* ≤ 0.05).

## 3. Results

**Figure 1:**
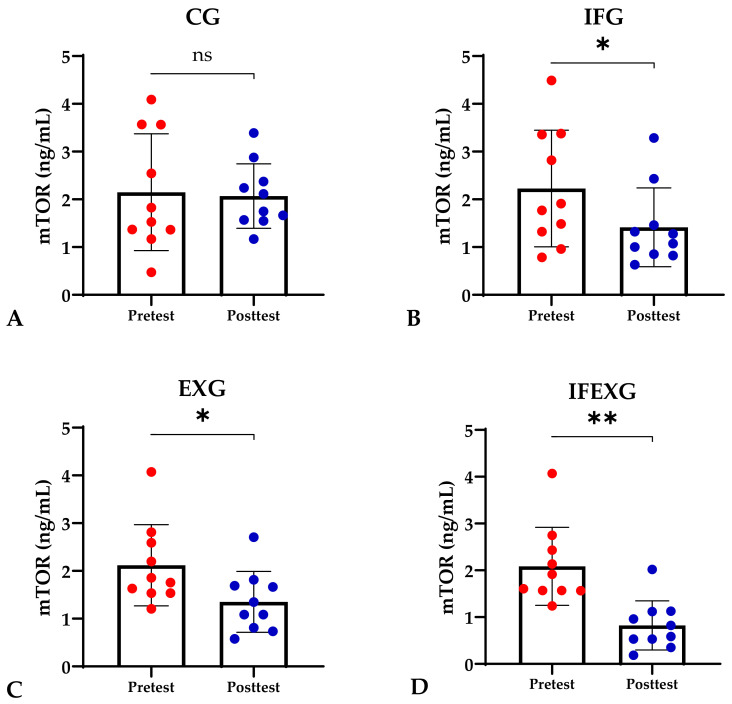
Differences in mTOR levels (ng/mL) between pretest and posttest in the four groups. (**A**) Control group (CG). (**B**) Intermittent fasting group (IFG). (**C**) Aerobic exercise group (EXG). (**D**) Combined aerobic exercise with intermittent fasting (IFEXG). Representative of data with mean ± SD. *p*-value was obtained by paired sample *t*-test. (ns) Not significant (*p* ≥ 0.05). (*) Significant at pretest (*p* ≤ 0.05). (**) Significant at pretest (*p* ≤ 0.001).

**Figure 2:**
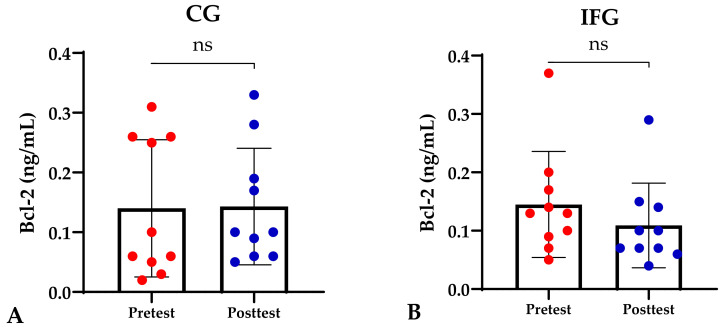

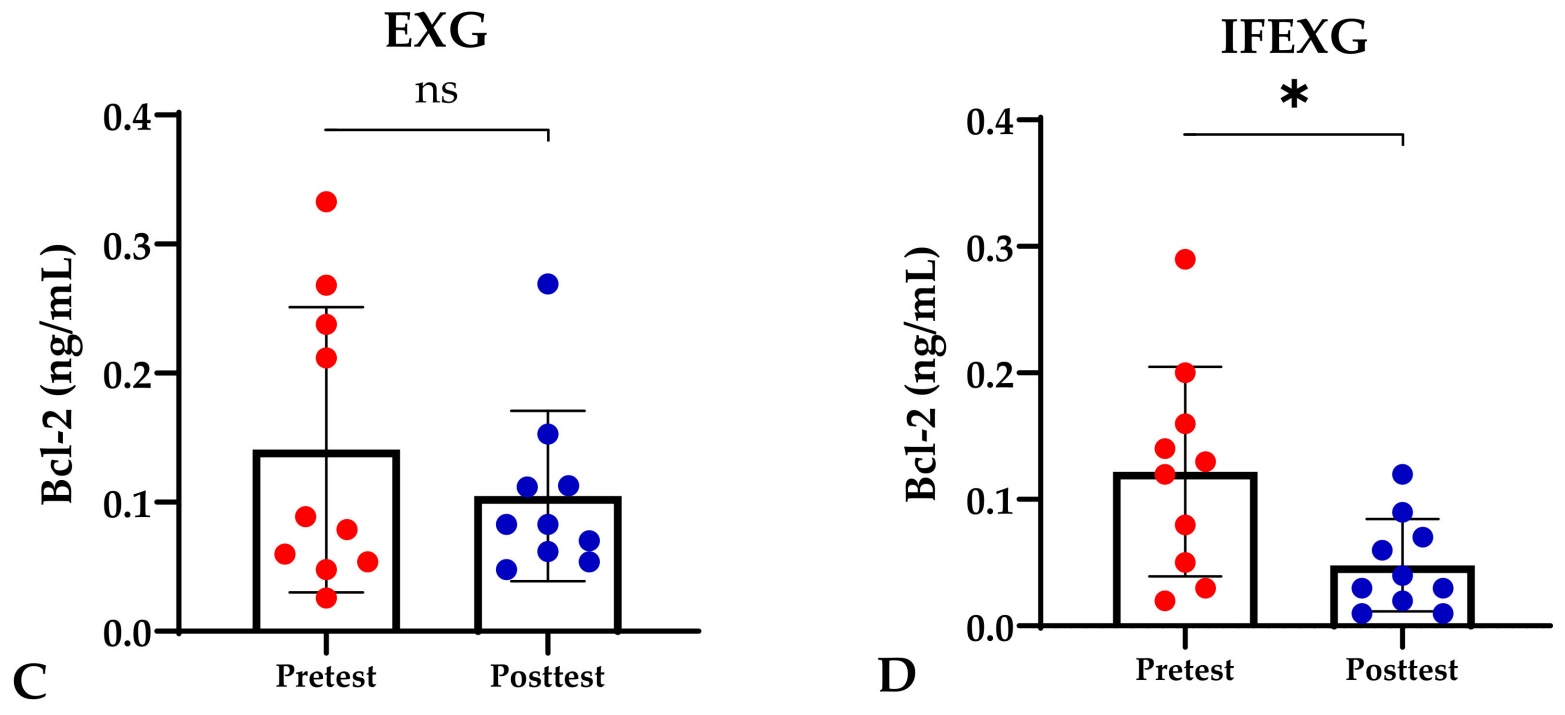
Differences in Bcl-2 levels (ng/mL) between pretest and posttest in the four groups. Representative of data with mean ± SD. (**A**) Control group (CG). (**B**) Intermittent fasting group (IFG). (**C**) Aerobic exercise group (EXG). (**D**) Combined aerobic exercise with intermittent fasting (IFEXG). *p*-value was obtained by Wilcoxon Signed Rank Test. (ns) Not significant (*p* ≥ 0.05). (*) Significant at pretest (*p* ≤ 0.05).

Based on the study results, no characteristics of the study subjects showed significant differences in the variables for each group (*p* ≥ 0.05), which are presented in Table 1. In this study, it was found that the IFG, EXG, and IFEXG interventions all significantly reduced mTOR levels. When reviewing the pre–post mTOR levels of the three intervention groups, IFEXG had a greater reduction than IFG and EXG (Figure 1). Meanwhile, Bcl-2 levels only decreased significantly in IFEXG (Figure 2). A comparison of mTOR and Bcl-2 levels between groups (CG vs. IFG vs. EXG vs. IFEXG) is shown in Table 2. Then, the results of our analysis of the association of the delta (∆) values for mTOR and Bcl-2 with body fat and metabolic age are shown in Table 3 and Table 4.

## 4. Discussion

Based on our study results, the combination of IF and aerobic exercise decreased mTOR levels in obese females significantly. Accordingly, in previous studies, it was shown that both IF and aerobic exercise were beneficial in reducing mTOR levels, mainly in vitro [33,34,35,36,37,38]. In accordance with our hypothesis, the present study showed that a combination of IF and aerobic exercise had a significant effect in reducing mTOR levels among obese females. However, there is still limited evidence regarding the effect of IF and aerobic exercise in reducing human mTOR levels, especially in obese females, through IF and aerobic exercise, and thus their potential to be used as a combined obesity treatment [39]. Different findings, from a study conducted in murine models, showed that only IF could downregulate the mTOR signaling pathway, and the addition of exercise would reactivate the mTOR signaling pathway in spite of this [40].

Physiologically, food intake increases blood glucose levels for the main energy source. mTOR is known to have an effect as a metabolic energy sensor that can integrate the variations in nutrient serum levels with endocrine responses [41]. During the feeding state, there will be an increase in the secretion of anorexigenic hormones including leptin and insulin, which leads to the inactivation of AMP-activated protein kinase (AMPK) in the hypothalamus and an increase in mTOR expression [42,43,44]. Increased mTOR will later promote cell growth by activating anabolic processes, stimulating energy storage in tissues, and preventing catabolism [45,46,47]. In a pathological state, excessive energy intake may lead to metabolic disturbance and overactivation of mTOR, thus increasing adipogenesis and fat storage, which contribute to obesity [48,49].

The main mechanism of IF is inducing a metabolic switch between the feeding and fasting states. Decreased carbohydrate intake during fasting results in decreased glucose levels and depletion of liver glycogen, and thus increased fat and protein metabolism as forms of compensation to fulfill the energy needs. Fatty acids released from adipose tissue will undergo β-oxidation in the liver and there will be an elevation of ketone bodies (β-hydroxybutyrate) for the energy source [50]. During this state, drastic drops in serum insulin levels will occur, leading to the activation of sirtuins and the AMPK pathway in the hypothalamus and inactivation of the mTOR pathway [51,52]. Fasting states also stimulate ghrelin secretion to induce the hunger sensation, and this will activate AMPK phosphorylation and inactivate the mTOR pathway [53]. These changes in AMPK and mTOR signaling are important in inhibiting FOXO-dependent gene transcription, resulting in the induction of autophagy and oxidative defense mechanisms [54]. In vitro studies have shown direct effect of IF in decreasing mTOR expression, thus promoting autophagy and rejuvenation, improving the cellular stress adaptation against ROS generation, improving the mitochondrial biogenesis function, and decreasing inflammation and DNA damage [55,56,57]. Through these metabolism changes, IF also provides benefits in improving several metabolic parameters, such as reducing fat mass, the total percentage of body fat, body weight, and the body mass index, and increasing the fat-free mass [39,58]. As shown in our study results, IF significantly decreases the body weight and fat mass in obese females.

Aerobic exercise was defined as any activity that used large muscle groups that relied on aerobic metabolism to form adenosine triphosphate (ATP) as the main energy source for the body. Aerobic activities increase energy consumption, which stimulates catabolism of the body reserves for ATP synthesis, instead of increasing the calorie intake for compensation [59,60]. During low- and mid-intensity aerobic exercise, the body increases fat usage for metabolism, while during high-intensity exercise, carbohydrates are used as the main energy source [61]. Similar to IF, aerobic exercise also affects mTOR via AMPK, while during exercise, ATP depletion will occur and induce the activation of AMPK mediated by liver kinase B1 (LKB1). The rise in AMPK levels will downregulate the mTOR levels, later inducing cell autophagy [24,62]. It is also known that aerobic exercise is the most effective type of exercise for treating obesity [63]. Previous studies showed that aerobic exercise effectively improved the body composition, marked by a reduced body fat mass, body weight, and BMI, and increased fat-free mass in the obese population [64,65,66,67]. Accordingly, in the present study, aerobic exercise decreased the body weight, BMI, and fat mass in obese females.

Other findings from our study showed that IF, aerobic exercise, and a combination of IF and aerobic exercise did not significantly decrease Bcl-2 levels in obese females. It remains unclear whether IF and aerobic exercise can regulate Bcl-2 levels. A previous study showed that Bcl-2 gene expression in the ventricular muscles of the aerobic-exercise-trained group was not significantly different compared to the non-intervention group [68]. Another study on fifteen weeks of swimming proved that exercise did not affect Bcl-2 levels [69]. However, a recent study by Koohshoori et al. demonstrated that Bcl-2 levels decreased after 4 weeks of aerobic exercise intervention [19]. Likewise, with IF, calorie restriction proved to decrease the Bcl-2/Bax ratio [70]. However, on the contrary, intermittent fasting upregulated the expression of other anti-apoptotic factors such as Akt and Bcl-2 [71].

Bcl-2 is the most characteristic anti-apoptotic protein [17]. Apoptosis is necessary to maintain tissue homeostasis, kill damaged or unwanted cells, and ensure proper development [72]. Bcl-2 works by blocking pro-apoptotic proteins released from mitochondria into the cytoplasm, such as c-cytochrome [17]. By regulating apoptosis, Bcl-2 helps cells respond appropriately to stress and prevents unnecessary cell death [73]. Bcl-2 also plays a role in stabilizing the outer mitochondrial membrane and preventing its permeabilization [74]. Dysregulation of Bcl-2 expression can lead to prolonged cell survival, thereby contributing to conditions such as cancer [75].

Conversely, Bcl-2 increased at the top of colon crypts in women and decreased in men after 12 months of moderate-to-vigorous exercise. However, the difference in effect by gender remains unknown [76]. A previous study proved that Bcl-2 gene expression in young and old cardiac muscle increased after four weeks of high-intensity interval training [77]. Exercise intervention on a high-fat diet was also proven to increase Bcl-2 levels significantly compared to the group that only received a high-fat diet. This suggests that 12 weeks of aerobic exercise can protect against mitochondria-mediated early apoptotic signals caused by obesity [78]. After 16 h of spontaneous wheel running, the Bcl-2/Bax ratio in skeletal muscles in normal healthy animals was proven to decrease [79]. Exercise effectively prevents cardiac apoptosis through the mitochondrial pathway by reducing ROS to a certain extent and preventing the release of mitochondrial cytochrome C [80]. Based on these different results, it can be surmised that Bcl-2 levels do not always show the same response pattern to physical exercise. However, there is limited evidence regarding the effects of intermittent fasting and exercise on Bcl-2 levels in obese females.

As mentioned before, both aerobic exercise and IF provide potential mechanisms for achieving better outcomes in mTOR levels, Bcl-2 levels, and other metabolic parameters in obese females, so their combination should have a synergistic effect. To the best of our knowledge, our study is the first to have evaluated the aerobic exercise and IF combined effect on the mTOR and Bcl-2 levels in obese females, and thus there is still limited evidence regarding this phenomenon. However, our findings revealed that combined aerobic exercise and IF could decrease mTOR levels, which indicates that this combination may help regulate cellular autophagy and physiological homeostasis, thereby potentially preventing metabolic disorders, aging, neurodegeneration, and cancer [81]. In line with our study, existing evidence also shows that combined aerobic exercise and IF improves metabolic parameters, such as decreased body weight and fat mass, enhanced insulin sensitivity (which could prevent diabetes, aging, and age-related disease), and increased cardiorespiratory fitness [82]. Thus, the combination of aerobic exercise and IF may offer a promising non-pharmacological treatment for obesity and prevent its related diseases.

The key limitations in this research include (a) its time-limited feeding protocol, which still needs to be further developed; even though this study used five days/week to carry out IF, the intake requirements were not adjusted to individual dietary needs in terms of quality or quantity, which should be monitored in the future to minimize bias in the research results. (b) The duration of the intervention in this study was only two weeks, and an improvement would be to see the impact of a combination of long-term fasting and exercise, so further research is needed. (c) This research was limited to the obese female population, and further research needs to be carried out in the male population, considering that obesity can also occur in men, to produce generalizable results.

## 5. Conclusions

The findings of this study were that all three types of intervention reduced mTOR levels. However, among the three interventions, the combined intermittent fasting and exercise group (IFEXG) received the optimal program for preventing aging by lowering mTOR, Bcl-2, body fat, and metabolic age levels. Interestingly, this study also found a positive relationship between reducing mTOR and Bcl-2 levels and reducing body fat and metabolic age. Meanwhile, a negative relationship was found between decreasing mTOR and Bcl-2 levels and increasing muscle mass.

## Figures and Tables

**Table 1:** Characteristics of the study population at baseline.

Parameters	(CG; n = 10)	(IFG; n = 10)	(EXG; n = 10)	(IFEXG; n = 10)	*p*-Value
Age (yrs)	23.90 ± 0.99	24.20 ± 2.69	23.30 ± 1.57	22.90 ± 1.59	0.390 †
SBP (mmHg)	111.45 ± 5.86	116.65 ± 12.37	119.95 ± 15.65	116.20 ± 3.43	0.360 †
DBP (mmHg)	67.68 ± 3.24	73.95 ± 8.55	75.30 ± 8.44	74.50 ± 7.13	0.086 †
HR (bpm)	76.80 ± 7.87	81.50 ± 5.72	79.30 ± 10.94	75.60 ± 4.79	0.336 †
SpO_2_ (%)	98.00 ± 1.05	98.80 ± 0.42	97.80 ± 1.23	98.40 ± 0.69	0.085 †
BT (°C)	35.96 ± 0.83	35.87 ± 0.58	35.58 ± 0.80	36.22 ± 0.67	0.285 †
FBG (mg/dL)	95.60 ± 2.50	94.90 ± 2.96	94.60 ± 2.01	95.60 ± 2.67	0.761 †
Hb (g/dL)	14.27 ± 2.02	13.81 ± 1.29	13.78 ± 1.50	14.04 ± 1.63	0.899 †
Height (m)	1.55 ± 0.03	1.57 ± 0.05	1.57 ± 0.06	1.55 ± 0.04	0.566 †
Weight (kg)	69.54 ± 6.10	75.22 ± 13.04	70.10 ± 9.88	76.26 ± 10.21	0.337 †
BMI (kg/m^2^)	29.19 ± 2.49	31.22 ± 5.01	28.36 ± 2.87	31.57 ± 3.61	0.157 †
FAT (%)	44.24 ± 2.66	45.84 ± 4.15	43.56 ± 3.54	47.21 ± 3.82	0.119 †
FM (kg)	31.72 ± 3.68	34.93 ± 9.11	30.78 ± 6.52	36.30 ± 7.49	0.260 †
FFM (kg)	38.65 ± 2.30	40.29 ± 4.31	39.32 ± 3.78	39.96 ± 3.10	0.725 †
MM (kg)	36.44 ± 2.09	37.94 ± 3.93	37.05 ± 3.47	37.62 ± 2.83	0.727 †
TBW (%)	28.42 ± 2.52	30.43 ± 5.68	28.83 ± 3.49	29.73 ± 3.14	0.659 †
TBW (kg)	41.03 ± 3.75	40.63 ± 2.53	41.27 ± 2.53	39.18 ± 2.96	0.411 †
BM (kg)	2.21 ± 0.21	2.35 ± 0.39	2.27 ± 0.32	2.34 ± 0.28	0.708 †
BMR (kcal)	1268.30 ± 80.96	1336.20 ± 168.73	1285.10 ± 146.22	1338.80 ± 125.68	0.553 †
MA (yrs)	48.70 ± 0.95	49.10 ± 2.88	48.90 ± 2.73	48.10 ± 2.18	0.790 †

Description: SBP: systolic blood pressure; DBP: diastolic blood pressure; HR: heart rate; SpO_2_: oxygen saturation; BT: body temperature; FBG: fasting blood glucose; Hb: hemoglobin; BMI: body mass index; FAT: percentage of body fat; FM: fat mass; FFM: free fat mass; MM: muscle mass; TBW: total body water; BM: bone mass; BMR: basal metabolic rate; MA: metabolic age. Control group (CG). Intermittent fasting group (IFG). Aerobic exercise group (EXG). Combined aerobic exercise with intermittent fasting (IFEXG). Representative of data with mean ± SD. (†) *p*-value was obtained by one-way ANOVA.

**Table 2:** Differences in mTOR, Bcl-2, body fat, and metabolic age between groups (CG vs. IFG vs. EXG vs. IFEXG).

Assessment	(CG; n = 10)	(IFG; n = 10)	(EXG; n = 10)	(IFEXG; n = 10)	*p*-Value
Pre-mTOR (ng/mL)	2.15 ± 1.22	2.23 ± 1.22	2.12 ± 0.85	2.09 ± 0.83	0.992 †
Post-mTOR (ng/mL)	2.07 ± 0.68	1.42 ± 0.83 *	1.36 ± 0.64 *	0.82 ± 0.53 *	0.003 †
∆-mTOR (ng/mL)	−0.08 ± 1.33	−0.81 ± 0.97	−0.76 ± 0.59	−1.26 ± 0.79 *	0.047 †
Pre-Bcl-2 (ng/mL)	0.14 ± 0.12	0.15 ± 0.10	0.14 ± 0.11	0.12 ± 0.08	0.932 ≠
Post-Bcl-2 (ng/mL)	0.14 ± 0.16	0.11 ± 0.07	0.11 ± 0.07	0.05 ± 0.04 *^$	0.015 ≠
∆-Bcl-2 (ng/mL)	0.01 ± 0.17	−0.04 ± 0.14	−0.04 ± 0.15	−0.07 ± 0.09	0.676 ≠
∆-Weight (kg)	0.75 ± 0.87	−0.59 ± 1.13 *	−0.78 ± 0.58 *	−1.10 ± 0.42 *	0.001 †
∆-BMI (kg/m^2^)	0.71 ± 1.25	−0.25 ± 0.47	−0.32 ± 0.24	−0.46 ± 0.19	0.002 †
∆-FAT (%)	0.58 ± 0.50	−0.30 ± 0.71	−0.96 ± 1.21	−1.31 ± 0.38 *^	0.001 †
∆-FM (kg)	0.42 ± 0.43	−0.49 ± 0.88 *	−0.70 ± 1.03 *	−1.16 ± 0.21 *^	0.001 †
∆-MM (kg)	−0.14 ± 0.88	0.39 ± 0.55	0.59 ± 0.69 *	1.15 ± 0.33 *^	0.001 †
∆-MA (yrs)	0.20 ± 0.63	−0.60 ± 1.27	−0.70 ± 1.57	−1.40 ± 0.69 *	0.026 †

Representative of data with mean ± SD. (†) *p*-value was obtained by one-way ANOVA and LSD post hoc test. (≠) *p*-value was obtained by Kruskal–Wallis test and Mann–Whitney U test. (*) Significant at CG (*p* ≤ 0.05). (^) Significant at IFG (*p* ≤ 0.05). ($) Significant at EXG (*p* ≤ 0.05).

**Table 3:** The association between delta (∆) of mTOR and Bcl-2 with body fat and metabolic age.

Parameters	∆-mTOR (ng/mL)		∆-Bcl-2 (ng/mL)	
	*r*	*p*-Value	*r*	*p*-Value
∆-Weight (kg)	0.315 *	0.048	0.397 *	0.023
∆-BMI (kg/m^2^)	0.537 **	*p* ≤ 0.001	0.331 *	0.037
∆-FAT (%)	0.316 *	0.047	0.369 *	0.044
∆-FM (kg)	0.358 *	0.023	0.367 *	0.035
∆-MM (kg)	−0.418 **	*p* ≤ 0.001	−0.501 **	*p* ≤ 0.001
∆-MA (yrs)	0.500 **	*p* ≤ 0.001	0.736**	*p* ≤ 0.001

* Significant with *p* ≤ 0.05. ** Significant with *p* ≤ 0.001.

**Table 4:** The association between metabolic age and body fat.

Parameters	∆-MA (yrs)	
	*r*	*p*-Value
∆-FAT (%)	0.523 **	*p* ≤ 0.001
∆-FM (kg)	0.514 **	*p* ≤ 0.001
∆-MM (kg)	−0.529 **	*p* ≤ 0.001

** Significant with *p* ≤ 0.001.

## Data Availability

The data presented in this study are available upon request from the corresponding author.
